# Promoting mental health in migrants: a GHQ12-evaluation of a community health program in Sweden

**DOI:** 10.1186/s12889-021-10284-z

**Published:** 2021-02-02

**Authors:** Olof Wrede, Jesper Löve, Junmei Miao Jonasson, Mamtuti Panneh, Gunilla Priebe

**Affiliations:** 1 Crisis and Trauma Unit, Region Västra Götaland, Gothenburg, Sweden; 2 School of Public Health and Community Medicine, Institute of Medicine, Sahlgrenska Academy, University of Gothenburg, Gothenburg, Sweden

**Keywords:** Mental health, Mental disorders, Asylum seekers, Newly arrived immigrants, Health promotion program, GHQ12

## Abstract

**Background:**

Research increasingly highlight post-migration factors for migrants’ mental health status. We investigated the association between participation in a health promotion program and changes in migrants’ mental health, and if socio-demographic factors and length of time in the new home country, Sweden, influenced a potential association.

**Methods:**

A five-week health promotion program named ‘Hälsostöd’ [Health Support], led by community health workers, was offered to migrants, primarily asylum seekers and newly arrived immigrants (*N* = 202). The framework for the program was salutogenic psycho-education, which focused on health effects of migration experiences, lifestyle and health, and the health care system. Mental health was measured at the start and end of the program. We analysed this follow up by using the recommended clinical cut off (i.e. > 11 of maximum 36, with higher scores indicating possible mental illness) in the 12- item version of the General Health Questionnaire (GHQ12). Chi Square test was used to analyse statistical significance of changes, and multinomial logistic regression to analyse associations to sociodemographic factors and length of stay in Sweden.

**Results:**

The number of participants scoring above the clinical cut off after participation in the program (*N* = 79, 39.1%) was lower compared to the corresponding number before participation (*N* = 111, 55.0%), Chi Square = 10.17, *p* < .001. The majority of the participants had no change 72.3 (*N* = 146), 21.8% (*N* = 44) had a positive change, yet 5.9% (*N* = 12) had a negative change, compared to before participation in the program. None of the investigated sociodemographic factors showed to significantly influence the association. Length of stay in Sweden was trending, with participants with longer stay being more likely to have possible mental illness.

**Conclusion:**

We conclude that psycho-educative programs, similar to ‘Hälsostöd’, have potential for promoting asylum seekers’ and newly arrived immigrants’ mental health as the evaluation showed a considerable number of positive changes in participants. The result suggests the importance of offering immigrants health promotive programs in close connection with arrival to the new home country. Future research should clarify under what circumstances sociodemographic factors influence the effects of such programs.

## Background

Between 2014 and 2017, Europe experienced an increased number of asylum seekers, and Sweden, specifically, received the most asylum applications ever noted in 2016 [1, 2]. A large proportion came from conflict areas such as Syria, Ethiopia, Eritrea, Congo, Afghanisation, and Iraq [3]. The health risks within this group vary greatly depending on, among other things, the type of conflict related experiences and the living conditions in refugee camps. Consequently, the WHO Regional office for Europe has emphasized refugee and migrant health as a priority area [4].

Existing studies indicate substantial levels of mental distress in this group [5–8], with several studies repeatedly recording higher rates of non-psychotic psychiatric disorders like Post-Traumatic Stress Disorder (PTSD), depression and anxiety as well as low levels of subjective mental well-being [8–10]. In Sweden, a population-based prospective cohort study showed a 33% increased risk of hospitalized depressive disorders in foreign-born compared to Swedish-born individuals [11]. A higher prevalence of PTSD, suicide, and psychotic disorders among migrants, compared to native Swedes has also been reported [12]. The causes of these adversities are multifaceted and include traumatic experiences both before and during the migration process. Increasingly, researchers also highlight post-migration factors for the prevalence of mental disorders [8, 10, 13, 14]. Such post-migration factors may include chronic stress in a new host country related to family members well-being [15], uncertainty regarding residence permit [16], a new language and social norms [9, 10, 17]. However, researchers have noted that the heterogeneity within this group makes it difficult to draw any all-embracing conclusions on health risks and appropriate interventions (e.g. [6, 10]).

A recent review concluded that in epidemiological studies on refugees the focus on PTSD needs to be accompanied by an increased focus on common mental disorders, e.g., depression and anxiety [18]. The same review also concluded that there is a need for studies assessing the efficacy of psychosocial interventions in migrants [18]. Due to the heterogeneity of this group, there is also a need to further investigate the associations between sociodemographic factors as well as the changes in participants’ mental health before and after taking part in psycho-educative programs. Among the few existing studies of such correlations, age and years in the new country of residence have been shown to relate to migrants’ mortality ratios as compared to native-born. For example, a U-shape pattern was observed between migrant’s age and mortality, with excess mortality at young age, lower mortality at adult age and a similar mortality with natives at older age [19]. A Swedish study showed that refugees who resided in Sweden for ten years or more were more likely to experience psychological distress, compared to those who arrived more recently [20]. Other studies suggest granted residence permit as positively associated with refugees’ mental health [16, 21].

A societal response to mitigate health risks in this group includes a strengthened focus on tailored interventions with, for example, trauma focused cognitive behavioural therapy and multidisciplinary treatments [10, 17, 22–24]. In reviews of such interventions [ibid.], authors conclude that long-term psycho-educational interventions and field oriented, pragmatic and multidisciplinary approaches are needed to further improve refugee’s mental health.

Interventions that focus on mental disorders in migrants with refugee background comprise both clinical and health promotive interventions. Where more therapeutic interventions in clinical settings focus on severe conditions like PTSD [24], health promotive interventions rather focus on increasing subjective well-being and preventing common mental disorders (e.g. depression and anxiety). Such salutogenic interventions, often depart from the concepts of ‘sense of coherence’ [25] and ‘coping’ [26], and aim to strengthen the participants’ health literacy, i.e. their ability to understand and beneficially handle their own living situation [27].

With the goal to support the considerable number of asylum seekers arriving in Sweden 2016, the Swedish government expanded existing mental health promotive programs and developed new ones in line with the salutogenic theories mentioned above. One of the most ambitious projects was ‘Hälsa i Sverige’ [Health in Sweden] [28], a national program consisting of education for health care staff, health care information directed at immigrants, and ‘Hälsostöd’ [Health Support], a five-session psycho-educative program aiming to promote immigrant’s mental health. Of the various components of the ‘Hälsa i Sverige’-project, the authorities within Region Västra Götaland decided to primarily focus on ‘Hälsostöd’ (see Method for details).

### Study aim and objectives

The aim of the current study was to investigate the effectiveness of the ‘Hälsostöd’ program on participants’ mental health status. The objectives were to (1) assess the association between partaking in the ‘Hälsostöd’ program and changes in participants’ mental health, and (2) to assess if sociodemographic factors and time in Sweden influenced such association.

## Methods

### Study setting and population

The ‘Hälsostöd’ program was initiated in May 2017 in Region Västra Götaland, Sweden. At the time, 16,043 migrants were registered at the Migration Agency in this region (4938 women and 11,105 men). Anyone with a migrant background was welcomed to participate. However, as the program started in response to the large influx of refugees in this region, the main target groups were asylum seekers and newly arrived immigrants with milder mental health problems.

Data was collected for this evaluation study between September 2017 and September 2018. At the first measure point, i.e. just before taking part in the first ‘Hälsostöd’ session, 496 participants completed the questionnaire. Although the records of the total number of attendees initiating the program were incomplete, the program staff estimated that those who completed the questionnaire, represented approximately 90% of the total attendees. Out of these 496, 319 also completed the study questionnaire after the program. Consequently, the study sample for the evaluation was 319 ‘Hälsostöd’ participants (see Table 1 for demographics). This means that 177 dropped out between the first and second data collection, equalling a 64% response rate. Most of the dropouts were due to the target group’s mobility, i.e. relocation to other geographical areas due to changes in the organisation of asylum seekers’ accommodation or a decision on the asylum application (positive or negative).

**Table 1:** Characteristics of study samples (T1 vs T1 plus T2)

Demographic factor	T1		T1 plus T2 (final study sample)	
	Total N	%	Total N	%
**Native language**				
Arabic	283	60.3	156	77.2
Dari	186	39.7	46	22.8
**Gender**				
Male	222	47.3	90	44.6
Female	238	50.7	109	54
Others	2	0.4	–	–
Missing^a^	7	1.5	3	1.5
**Residence status**				
Asylum seeker	127	27.1	40	19.8
Residence permit	331	70.6	162	80.2
Others	4	0.9	–	–
Missing^a^	7	1.5	–	–
**Education (years)**				
0–4	94	20	29	14.4
5–9	125	26.7	57	28.2
> 10 years	230	49	111	55
Missing^a^	20	4.3	5	2.5
**Years in Sweden**				
< 1	77	16.4	36	17.8
1–2	89	19	38	18.8
> 2	294	62.7	126	62.4
Missing	9	1.9	2	1.0
**Age**				
Mean = 39.63, Standard Deviation = 12.23, Range = 19–76				

^a^ Excluded from regression analyses

The aim of the ‘Hälsostöd’ program, which consisted of five 2–3 h sessions (held once a week), is described in its manual to: “strengthen a positive development of mental health through providing the individual with tools for coping with minor mental health issues”. The sessions were led by community health workers, so called health communicators, who themselves had migrant backgrounds, some form of health care education (e.g., nursing, pharmacy), additional training in group dynamics and the ‘Hälsostöd’ content.

Around eight participants were included in each ‘Hälsostöd’ group. The program was held in the participants’ native language, in public venues such as libraries and immigration centres, and was free of charge. Each session had a specific topic: introduction; possible health effects of migration; ways to identify and prevent stress and depression; the health effects of lifestyle factors; and the organization of the Swedish health care system. A strong emphasis was placed on creating a safe space where participants could talk freely about their experiences and current situation. The role of the health communicators was to facilitate a trusting atmosphere, to function as a bridge between the participants’ previous and current environment, and to adapt the health messages to the respective cohort’s level of education, needs, and interests. The intent was to encourage and frame empowering discussions around set health topics rather than to mediate unidirectional information. In sum, the program can be described as combining several components of the salutogenic theory, a theory that highlights promotion of mental health through increased health literacy and ‘sense of coherence’ [25], and the opportunity to, together with peers, elaborate on personal experiences [27].

### Data collection and study procedure

The GHQ12, a shortened version of the General Health Questionnaire, was developed to identify minor mental health problems, e.g., symptoms of depression and anxiety or other symptoms in need of further clinical assessment with the purpose of early identification of potential psychiatric disorder [29]. It was available in Arabic, but had to be translated (back-and-forth) for Dari and Somali. GHQ12 was chosen to evaluate this program as it has been validated for identifying milder mental health issues in many countries and populations [30–32], i.e. in relation to such a heterogeneous group that was the ‘Hälsostöd’ target group. In addition, GHQ12 is not niched on any specific diagnosis (such as e.g. PHQ9 for depression), and, as it is short and clear, it is suitable for a study population with varying levels of education and stress symptoms (e.g. compared to WHODAS, SRQ20). The Likert method was chosen for scoring GHQ as this method enabled comparisons between subgroups in this very diverse study population (see [33]). The use of a standardized cut off limit is useful for the assessment of need of health care. It was set to > 11, as recommended, where a higher score is understood as indicating mental health problems in need of further clinical investigation [30, 34].

As the study population is considered a hard-to-reach group, a decision was made to involve the health communicators in the recruitment to the study. To counteract possible disadvantages of not separating the study recruitment entirely from the program implementation, the last author trained the health communicators in research ethics and principles for scientific validity, including how it relates to the recruitment process. In addition, either the first or last author was initially present when the health communicators informed potential participants about the study and then followed up with supervision until the respective health communicator was considered to master the ethical, validity and administrative aspects of recruitment.

Written information about the study was distributed at health information sessions for newly arrived immigrants, at Integration centres’ pin boards and in Swedish classes for immigrants. Those who were interested in participating in the ‘Hälsostöd’ program and in the study were invited to receive oral information, by a health communicator, in their native language one hour before the first ‘Hälsostöd’ session. After the study information, those who agreed to participate signed an informed consent form (in Swedish plus native language), filled in the questionnaire (native language), enclosed it in an envelope and handed the sealed envelope to the health communicators’ team leader, who delivered it to the research team. Filling out the questionnaire was repeated after the fifth and final ‘Hälsostöd’ session.

### Variables

#### Outcome variables

In the present study, a positive change was defined as a decrease in GHQ12 score from > 11 before program participation to ≤11 after participation. The effect of participation in the ‘Hälsostöd’ program has thus mainly been measured in relation to a clinically significant change (need/no need for further clinical investigation), i.e. not in relation to mean or total score change.

#### Covariate variables

The following sociodemographic factors were included (see Table 2): age (in years), gender (men, women, other); education (years of education: 0–4, 5–9 or > 10 years); years in Sweden (< 1, 1–2, > 2 years); residence status (asylum seeker, granted residence permit, other).

**Table 2:** Odds Ratios (95% CI) between covariates and outcome

Predictor	OR (95% CI)	
	Positive Change^**a**^	Negative Change^**a**^
**Age**	1.02 (0.99 to 1.05)	1.05 (0.99 to 1.11)
**Gender**^b^	0.67 (0.31 to 1.45)	0.70 (0.18 to 2.72)
**Education**		
0–4 years	1.79 (0.60 to 5.29)	1.37 (0.21 to 9.00)
5–9 years	1.00 (0.39 to 2.52)	1.66 (0.37 to 7.59)
> 10 years	Reference	Reference
**Years in Sweden**		
0–1 years	1.71 (0.64 to 4.59)	0.47 (0.05 to 4.25)
1–2 years	1.30 (0.48 to 3.51)	0.45 (0.05 to 3.99)
> 2 years	Reference	Reference
**Residence status**^c^	0.50 (0.17 to 1.51)	1.08 (0.18 to 6.48)

^a^ The reference group is No Change

^b^ Males coded as 1, females as 2

^c^ Asylum seeker coded as 1, granted residence permit as 2

### Statistical analyses

Both descriptive and inferential statistical analysis were conducted. The total numbers (N) and percentages (%) were used to present categorical variables. The means (M) and standard deviation (SD) were used to describe continuous variables. We calculated the mean and SD of GHQ12 score both at baseline and after program participation. The frequencies of dichotomous directions of changes in GHQ12 levels were calculated. The change outcome was coded in three categories (positive change; negative change; no change) using the recommended cut off score for clinically significant changes (i.e. > 11). Moreover, a Chi-square test (*p* < .001) was used to evaluate the differences in GHQ12 > 11 at baseline compared to after program participation.

The change categories (positive change, negative change, no change).were applied in multinomial logistic regression, which modelled the above presented predictors in relation to GHQ12 outcomes, for the purpose of answering the research question related to sociodemographic factors. The Somali and Persian groups were very small. Hence, we excluded this data from further analysis. Moreover, we defined a cut off for program attendance to participation in at least 60% of the program sessions (i.e. ≥3). This because participation in only the first and last session, in which measurements were administrated, would not give a fair picture of the influence of the program content. The mean attendance of the included group was 4.50 sessions. Finally, a conservative approach, to only include participants with no missing GHQ12 items, was chosen, as dealing with solid data is of particular importance in novel research areas. The final dataset included *N* = 202 cases (see Table 1 for demographics). IBM SPSS Statistics 25 was used as software for analyses.

## Results

Table 1 presents the baseline characteristics of those ‘Hälsostöd’ participants that were included in the analysis.

The descriptive analysis showed that the mean of GHQ12 score, for the study population, was higher before participation in ‘Hälsostöd’ (M = 12.74, SD = 6.83) compared to after (M = 10.83, SD = 6.15). In addition, a frequency analysis showed that 21.8% (*N* = 44) of the participants had a positive change, 5.9% (*N* = 12) had a negative change and 72.3% (*N* = 146) had no change compared to before the program. To investigate potential clinical relevance of the observed change, we also analysed the number of participants scoring > 11, i.e., the cut off indicating a need for clinical investigation, after participation compared to before. The number of participants scoring > 11 after participation was lower (*N* = 79, 39.1%) compared to the corresponding number before participation (*N* = 111, 55%), Chi Square = 10.17, *p* < .001). This result suggests that fewer participants had symptoms of adverse mental health after the program. Moreover, the majority did not deteriorate in mental health over the study period. The majority rated a median of 7-point decrease on the GHQ12 measure. In the negative change group, participants rated a median of 6-point increase on their GHQ12 score. No statistically significant associations between sociodemographic factors and changes in mental health before and after program participation were observed (Table 2).

## Discussion

This study investigated migrants, mainly asylum seekers’ and newly arrived immigrants’, self-reported mental health before and after participating in the five-week health promotive program ‘Hälsostöd’. The main result shows that the number of participants in need of further clinical investigation for mental illness was lower after the program compared to before. However, we did not observe any statistically significant association between investigated sociodemographic factors and outcome.

Psycho-educative programs that promote participants’ health literacy, i.e. their ability to understand, manage and control their health development and the situation they are in, arguably connect to Antonovsky’s theory of ‘sense of coherence’ (see [25]). According to the theory, a sense of coherence promotes people’s use of their own and the context’s resources when coping with stress and tension [27]. The increased sense of coherence following improved health literacy, an anticipated consequence of the program, is a possible explanation of the observed positive changes in participants’ GHQ12 scores. Although using a different scale (Kessler), an evaluation of a similar program aimed at Arab refugees found a comparable decrease in general mental illness [35]. Our results add to that finding by further underscoring the potential of psycho-educative health promotion programs on migrants’ mental health.

More so, efforts to bridge linguistic and cultural barriers between refugees and health workers have been revealed paramount in securing intervention effectiveness [27, 36–38]. In this program, the health communicators’ migrant background was intended to support trust, empathy, and connection with the target group [28]. Further research is needed on which aspects of health communicators’ profile are essential for the achievement of program objectives, yet the idea of identification connects to research on mechanisms behind positive effects of psychotherapy, which clearly underscores personal factors. Although empathy and identification are not enough to create clinical change, it is fundamental to human interaction and is described as key to support patients’ and health promotion program participants’ motivation towards co-creating positive change [39, 40].

Previous research has shown that post-migration factors in the new host country (e.g. waiting for a residence permit, un-employment, low socioeconomic position) impact and presuppose a deterioration in mental health [17]. For example, hazard risks for ICD-10 nervous and psychotic disorders steadily increases with each quarter of waiting time for asylum and residence decisions [16]. A similar development could therefore be expected for the participants in this program, which is why ‘No change’ in mental health potentially could be viewed as a positive outcome.

Our assumption that sociodemographic factors could moderate the influence of the program on participants’ mental health was not supported by the study result. None of the analysed sociodemographic factors (gender, education, age, years in Sweden, and residence status) showed any statistically significant associations with mental health outcomes. The effect of years in Sweden was trending, as participants with lesser years in Sweden to a higher degree reported positive change in mental health. We hypothesise that this trend could reflect the negative influence of long waiting for asylum decisions on applicants’ mental health [20, 22, 35]. Long waiting for asylum decisions applied to the majority of the participants in our study because of the intensified application numbers in Sweden at the time, which led to decisions taking up to several years.

A possible explanation of the null result is related to adaptation of the program content to participants’ different needs, as the pragmatism and flexibility of the person leading this kind of group sessions are highlighted as crucial for their quality (e.g. [22, 41]). It is possible that the health communicators in this program, to an unusually high degree, managed to satisfactorily meet the varying profiles and needs of the participants. If they managed, in accordance with program instructions, to create a trusted environment that provided room for the participants’ own migration narratives, and adapt the program content to the participants’ level of education and age, this could have levelled out the influence of demographic factors, rendering the ‘Hälsostöd’ sessions equally meaningful to all subgroups.

However, the null findings should be interpreted with caution and it is possible that larger sample sizes of each sub-group are needed to detect potential associations. Additionally, the study population in this study was more heterogeneous than in previous studies stating an association between sociodemographic factors and mental health outcomes (e.g. [11]), a fact that could have decreased their predictive value for the evaluation of this program.

### Limitations

A few important limitations should be noted. Firstly, for ethical and practical reasons, it was not feasible to include a control group. Consequently, conclusions about causality are not possible, even though mental health changes were observed in individuals and on group level. To enable comparison, future studies could try including a waiting list control, using the administration of self-reports on mental health before program participation, and/or additional measuring points, preferably with long-term follow-ups. The latter was not possible in this study because of limited project time frames and because many participants changed address or even left the country during the study. Digital solutions may enable ways to keep connections to participants in future studies. However, possible opportunities to include control groups and innovative ways of increasing the follow-up period should be balanced with ethical considerations due to the susceptibility of this group.

The dropout rate is a limitation, partly because it was especially high for particularly vulnerable groups, i.e. the Dari group and the asylum seeker group. Some of the increased difference between ‘asylum seekers’ and ‘residence permit’, between T1 and T2, reflect that a few participants received their residence permit during the program and some moved to other regions. However, some could also have dropped out because of a deteriorating mental health status, linked to their challenging situation. Taken together, the results of our study may not fully apply to Dari and asylum-seeking participants. Future studies should thus look closer on the health statuses of these groups in order to increase the understanding of what type of programs could be relevant for promoting mental health in groups in exceptionally difficult social circumstances. Hence, we encourage future evaluations of similar programs to specifically look at possible subgroup differences and timing of the program in relation to participants’ life premises, e.g. asylum status.

Another limitation of this study is the fact that the health communicators assisted in the information and recruitment to the study. We cannot exclude bias effects, particularly in relation to low educated groups needing extensive explanations for understanding e.g. the Likert scale logic. However, to make use of a trusted, native speaker, i.e. the health communicators, was in our judgment a necessary strategy to recruit participants to the study. In addition, our sample may be subjected to selection bias as participating in the ‘Hälsostöd’ program demanded a certain level of structure, energy, and social engagement over five weeks. As was mentioned above, members of the target group with deteriorating mental health status may have had less possibility to complete the program. Even so, several of the included study participants reported high GHQ12 scores at the interventions start, suggesting very poor mental health, but still fulfilled the five sessions, which contests a general selection bias. The lack of screening for mental disorders before the program start enabled participants on all levels of mental health to participate. Since the intervention was not designed to have an effect on more severe mental disorders, the inclusion of such cases in the study sample might have lowered the positive change in average score. Finally, the specific contextual situation of Sweden for asylum seekers and newly arrivals at the time possibly limits the generalization of the results to other settings and study populations.

## Conclusion

Altogether, our study suggests that migrants’, specifically asylum seekers and newly arrived immigrants’, mental health can benefit from participating in a salutogenic psycho-educative health promotion program lead by community health workers. This was shown by positive changes in participants’ GHQ12 scores, meaning a lowered proportion of individuals in need of further clinical investigation. Previous research suggests a need to further investigate association between sociodemographic factors and the mental health effect of certain interventions. However, this evaluation did not establish any such correlations which could be due to the health communicators ability to effectively adapt to each groups’ needs. Findings in this study suggest the importance of offering refugees and other immigrants tailored health promotive programs in close connection with arrival in the new home country. The provision of mental health and health literacy focused programs could help reduce the disproportionate health challenges faced by migrants and refugees.

## Data Availability

The datasets generated and/or analysed during the current study are not publicly available due to ethical and GDPR restrictions, but are available from the corresponding author on reasonable request.

## Abbreviations

GHQ12: General Health Questionnaire 12 items
PTSD: Post-Traumatic Stress Disorder
